# Outreach for Young Adult African Americans with Risk Factors for Stroke

**DOI:** 10.3928/24748307-20240220-01

**Published:** 2024-01

**Authors:** Iris Feinberg, Dawn M. Aycock, Elizabeth L. Tighe, Delaney Detamore

## Abstract

**Background:**

Research suggests that younger adult African American people (age 18–35 years) have more than double the risk of having a stroke than White people. Stroke risk education is lacking for this cohort; there is a dearth of materials that are targeted and focused for young adult African Americans. There is also little research on developing and testing age and culturally appropriate health literate materials that may help this population better understand personal risk factors for stroke.

**Objective:**

The aim of this study was to understand factors to guide creating and disseminating plain language health messages about stroke risk awareness among young adult African Americans.

**Methods:**

African American participants age 18 years and older completed an online survey (*N* = 413). Descriptive statistics, one-way analysis of variance, and two-step cluster analyses were used to evaluate stroke risk awareness, perceived risk of stroke, message creation factors, and online health information seeking behavior. Open-ended survey items described modifiable and non-modifiable reasons for perceived risk of stroke.

**Key Results:**

Participants reported differences on overall stroke risk factor awareness by perceived risk of stroke was significant (F[2, 409] = 4.91, *p* = .008) with the *very low/low* group (*M* = 1.66, *p* < .01), showing significantly lower overall stroke risk factor awareness compared to the *moderate* and *high/very high* groups. Both respondents who thought their stroke risk was *very low/low* and *moderate/high/very high* commented about family history (54.1% and 45.9%, respectively) as the reason and 88.2% of *very low/low* commented that they did not have risk factors for stroke because they were young. Cluster analysis indicated the Mostly Clear Preferences cluster was more likely to select *mostly/very* on positive, informational, and long-term messages and medical authority sources. The largest of three clusters reported medical sources as the highest rated source for both finding and trusting health information (47.2%, *n* = 195).

**Conclusion:**

Young adult African Americans have a scarce understanding of modifiable stroke risk factors; health education materials should focus on positive information messaging that shows a long-term result and is presented by a medical authority. We did not observe any age or sex differences among the data, which suggests different message modalities may not be needed. [***HLRP: Health Literacy Research and Practice*. 2024;8(1):e38–e46.**]

## Abstract (plain language summary)

**Plain Language Summary: **In this study, we collected data to create a targeted stroke risk awareness health education video for young African American adults (age 18 years and older). The video was based on analysis of data from 413 participants focusing on perception of stroke risk, stroke risk knowledge, as well as preference for message type, source credibility, and modality.

Stroke is a largely preventable disease, which makes the rising incidence in younger populations a major public health issue (Béjot et al., 2016). Despite the increase, awareness of cardiovascular and stroke risk factors among young adults is inadequate (Aycock, et al., 2023; Bucholz et al., 2018). The first step to addressing early onset stroke is increasing awareness. Research suggests that younger adult African Americans have more than double the risk of having a stroke (Kissela et al., 2012) and experience reduced functional outcomes when compared to White people (Jones et al., 2020). Common modifiable risk factors contributing to stroke in young adult African Americans include hypertension, diabetes, cigarette smoking (Aradine et al., 2022; Jones et al., 2020), and obesity (Mehndiratta et al., 2022). However, research demonstrates gaps between young adult African Americans perceived risk of stroke and their actual risk of stroke (Aycock et al., 2017; Aycock et al., 2023), which may hinder the adoption of risk reduction behaviors.

For health information on stroke risk to be effective, it must be accessible, understandable, and usable for its targeted audience. Differences exist between individuals in their levels of health knowledge, interest in health information, and health information-seeking behaviors. Age and culturally appropriate health communication grounded in the health literacy principle of being able to use health-related information to make informed decisions are needed to improve accurate stroke risk perception for young adult African Americans as there is a dearth of studies on understanding these barriers within African American communities (Muvuka et al., 2020; Spruill et al., 2015). Accessible, understandable, and usable health literate information that is targeted and focused may help young adult African Americans better understand personal risk factors for stroke and influence informed health behaviors.

Health messaging that is targeted to specific minority groups results in interventions with higher success rates than ones focused on the general population (Brewer et al., 2019; Vuong et al., 2020). Engaging the end user in materials development is a key step to ensure health education materials are meaningfully and culturally developed (Covello et al., 1987). Other factors such as message framing, source credibility, and delivery modality affect how health information is received and acted upon. Message framing is a way to tailor a message to a particular audience and has been successfully used in numerous health behavior change settings (O'Keefe & Jensen, 2007; Park et al., 2020) by presenting equal decision outcomes in various formats such as positive gain or loss framing. Individuals may also shape their health beliefs based on information from trusted sources, who display credibility through expertness and trustworthiness (Clayman et al., 2010). Establishing trust between the information source and target population is important to the success of health communication interventions (Wathen & Burkell, 2001; Wilkins, 2018).

Along with health literacy guidelines, text modality and visual format must be considered when developing health education materials (Meppelink et al., 2015). Studies suggest that health prevention messages delivered via video using a narrative (storytelling) format produce more significant persuasive effects (Shen et al., 2015). Further, research on multimedia learning shows that presenting visual animations with written text can create an adverse cognitive load, and as such, animations may be more beneficial when they co-occur with orally presented language than when they co-occur with texts (Mayer & Morenoo, 2010). A recent systematic review of eHealth interventions on lifestyle-related diseases such as stroke reports that methods such as videos, films, and movies are satisfactory to end users; however, there are little data on targeting text modality and visual format of preventive health messaging specifically to young adult African Americans (Aida et al., 2020).

The internet is the most often used source of health information seeking among adults in the United States; however, few studies focus specifically on where young adult African Americans search for or trust health information (Finney Rutten et al., 2019; Hills & Shah, 2020; Jackson et al., 2019). Web-based information sources present optimal dissemination channels of health information because the internet can be accessed any time and from almost anywhere; however, “the internet” is not just one source; rather, health information on the internet can come from a variety of channels including medical authorities, social media, news websites, and other places. According to the Pew Research Center (2021), in addition to ubiquitous use of YouTube and Facebook by young adults age 18 to 29 years (95% and 70%, respectively), Instagram and Snapchat are more commonly used by young adults than by older adults. African Americans of all ages indicate use of YouTube (84%), Facebook (74%), and Instagram (49%) as their most often viewed online platforms (Pew Research Center, 2021). What is less clear is trustworthiness of information seen through these various online platforms. For the African American community, there are deeply held beliefs about not trusting health providers or health information stemming from historical and current inherent racial bias within the health care system writ large (Kennedy et al., 2007; Miller & Miller, 2021). A recent meta-analysis shows that quality and trustworthiness were dominant predictors of seeking health information online for the general population; however, there is a gap in evaluating trust of health information among online sources such as Facebook, You-Tube, or Twitter (Wang et al., 2021), especially for young adult African Americans who may be at significant risk for stroke.

The goal of this study is to understand factors to guide creating and disseminating plain language health messages about stroke risk awareness among young adult African Americans age 18 to 30 years. Our study is guided by the following research questions:

1: Is there a relationship between perceived risk of stroke and awareness about stroke risk factors? Does that differ by sex or age?
2. What are the reasons people believe they are at low, moderate, or high risk for stroke?
3. What are the most important factors for creating a message (type of message, source credibility, modality)? Does that differ by sex or age?
4. Where do people look for and trust health information? Does that differ by sex or age?

## Methods

### Sample

Individuals age 18 to 30 years who live in Georgia and self-identify as African American were recruited using Qualtrics Research Services (QRS). The participant pool (*N* = 413) was stratified to mirror state-wide demographics of sex (52% female, 48% male) (US Census Bureau, 2021). These individuals were sent an email invitation or prompted on the survey platform to proceed with the survey; interested respondents clicked on a hyperlink to access the survey. Participants were incentivized with cash, gift cards, or retail store points according to their individual agreement with QRS. The study was approved by the Georgia State University Institutional Review Board and informed consent was obtained prior to data collection.

### Measures

We collected demographic information on age, sex, family history of stroke, and perception of stroke risk. Respondents were asked to rate their perception of stroke risk as *very low*/*low*/*moderate*/*high*/*very high* and to provide a qualitative response as to the reason they selected their option using a single-item measure that has been validated in this population (Aycock et al., 2019; Aycock et al., 2023). The stroke risk factor awareness (SRFA) measure consisted of seven items about different conditions or behaviors, all of which should be correctly identified as more or most risky based on stroke risk factors identified by the American Stroke Association (2023). Participants were asked to rate each factor using a five-point Likert-style scale from least to most risky. Each condition or behavior that was correctly rated *more* or *most* risky was assigned a value of 1; scores were summed to create the total SRFA score. Internal consistency reliability was measured by Cronbach's alpha with a coefficient alpha of .86.

Message type, source credibility, and delivery modality were measured using a four-point Likert-style scale. Participants were asked to rate each characteristic from *not at all* to *very* helpful. Message types were positive, negative, information only, short-term results, long-term results and included a descriptive example. Source credibility types were celebrity, medical authority, government organizations, and “people like me.” Modality types were live person action/talking, animated person action/talking, pictures with written words, and pictures with someone talking in the background. Health information seeking behavior was measured by respondents' answering *yes*/*no*/*don't know* for finding information and for trusting information; health information sources were social media (Facebook, Instagram, TikTok, Twitter, Reddit, YouTube), medical website (hospital, doctor office, clinic, WebMD, other medical sources), and other resources (internet news site, family/friends' emails). Internal consistency reliability was measured by Cronbach's alpha with a coefficient alpha of .87.

### Data Analyses

We used SPSS, Version 27 for all analyses (IBM Corp., 2020). Descriptive statistics (means, standard deviations, frequencies) are reported in **Tables 1** and **2**. For research question 1, we used a one-way ANOVA (analysis of variance) with follow-up Tukey corrected comparisons. For research questions 2 and 3, we conducted two-step cluster analyses with follow-up chi-square and *t*-test comparisons for potential demographic differences among clusters. We used NVivo (released in 2020) to analyze qualitative data on belief of stroke risk; we provide descriptive statistics and qualitative analysis.

**Table 1:**
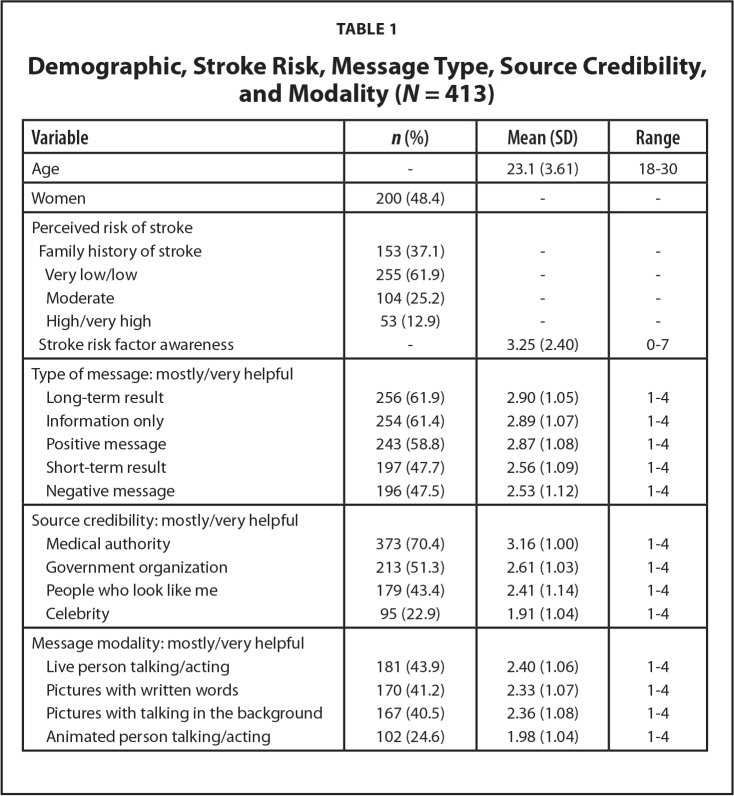
Demographic, Stroke Risk, Message Type, Source Credibility, and Modality (*N* = 413)

**Variable**	***n* (%)**	**Mean (SD)**	**Range**

Age	-	23.1 (3.61)	18–30

Women	200 (48.4)	-	-

Perceived risk of stroke			
Family history of stroke	153 (37.1)	-	-
Very low/low	255 (61.9)	-	-
Moderate	104 (25.2)	-	-
High/very high	53 (12.9)	-	-
Stroke risk factor awareness	-	3.25 (2.40)	0–7

Type of message: mostly/very helpful			
Long-term result	256 (61.9)	2.90 (1.05)	1–4
Information only	254 (61.4)	2.89 (1.07)	1–4
Positive message	243 (58.8)	2.87 (1.08)	1–4
Short-term result	197 (47.7)	2.56 (1.09)	1–4
Negative message	196 (47.5)	2.53 (1.12)	1–4

Source credibility: mostly/very helpful			
Medical authority	373 (70.4)	3.16 (1.00)	1–4
Government organization	213 (51.3)	2.61 (1.03)	1–4
People who look like me	179 (43.4)	2.41 (1.14)	1–4
Celebrity	95 (22.9)	1.91 (1.04)	1–4

Message modality: mostly/very helpful			
Live person talking/acting	181 (43.9)	2.40 (1.06)	1–4
Pictures with written words	170 (41.2)	2.33 (1.07)	1–4
Pictures with talking in the background	167 (40.5)	2.36 (1.08)	1–4
Animated person talking/acting	102 (24.6)	1.98 (1.04)	1–4

**Table 2:**
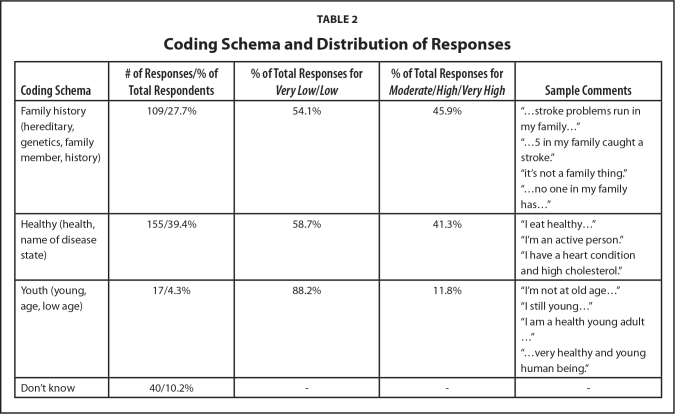
Coding Schema and Distribution of Responses

**Coding Schema**	**# of Responses/% of Total Respondents**	**% of Total Responses for *Very Low*/*Low***	**% of Total Responses for *Moderate*/*High*/*Very High***	**Sample Comments**

Family history (hereditary, genetics, family member, history)	109/27.7%	54.1%	45.9%	“…stroke problems run in my family…”
				“…5 in my family caught a stroke.”
				“it's not a family thing.”
				“…no one in my family has…”

Healthy (health, name of disease state)	155/39.4%	58.7%	41.3%	“I eat healthy…”
				“I'm an active person.”
				“I have a heart condition and high cholesterol.”

Youth (young, age, low age)	17/4.3%	88.2%	11.8%	“I'm not at old age…”
				“I still young…”
				“I am a health young adult …”
				“…very healthy and young human being.”

Don't know	40/10.2%	-	-	-

To address research question 2, we conducted a structured qualitative analysis to understand what reasons people cited for their perceived risk of stroke. We cleaned the data to remove comments that were not appropriate answers to the question (*n* = 12; e.g., “I see you today” or “I saw Singh and he was golfing”). A coding schema was developed according to organically occurring thematic constructs, which were *health*/*being healthy*/*health risk* (39.4% of all respondents mentioned), *family history of stroke* (27.7% of all respondents mentioned), *youth*/*young age* (4.3% of all respondents answered) and *don't know* (10.2% of all respondents). Two researchers (D.D., M.M.O.) and the principal investigator (I.Z.F.) reviewed the coding to ensure clarity prior to coding the transcriptions. The two researchers (D.D., M.M.O.)coded the same sets of files; results were reviewed by the principal investigator (I.Z.F.) to resolve discrepancies. There were five discrepancies to resolve; according to Cohen's κ, there was strong agreement between the two researchers (D.D., M.M.O.), *κ* = .875, 95% confidence interval [.570, .946], *p* < .001.

## Results

A total of 729 people accepted an invitation to the survey, with 413 meeting purposive stratification sampling criteria for a 56.7% respondent rate. All 413 fully completed the study with a 100% response rate. Less than one-half identified as women (48.4%). All respondents were African American, and age ranged from 18 to 30 years with an average of 23.12 years (standard deviation [*SD*] = 3.61). Most participants perceived their risk of stroke as *very low* or *low* (61.9%) and stroke risk awareness was also *low*. Participants accurately rated an average of 3.25 (*SD* = 2.40) out of 7 risk factors (**Table 1**). Scores for *mostly*/*very helpful* for message type, source credibility, and modality are shown in **Table 1**.

Research Question 1: Is there a relationship between perceived risk of stroke and awareness about stroke risk factors? Does that differ by sex or age?

To address research question 1, a one-way ANOVA was conducted to examine differences on overall stroke risk factor awareness by perceived risk of stroke groups (1 = *very low*/*low*; 2 = *moderate*; 3 = *high*/*very high*). The overall ANOVA was significant (F[2, 409] = 4.91, *p* = .008). Follow-up Tukey comparisons indicated that the *very low*/*low* group (*M* = 1.66, *p* < .01) had significantly lower overall stroke risk factor awareness compared to the moderate (*M* = 2.26, *p* = .030) and *high*/*very high* perceived risk of stroke groups (*M* = 2.38, *p* = .049). There were no significant differences between the *moderate* and *high*/*very high* groups (*p* = .937). There was no difference by sex or age.

Research Question 2: What are the reasons people believe they are at low, moderate, or high risk for stroke?

Both respondents who thought their stoke risk was *very low*/*low* and *moderate*/*high*/*very high* commented about family history (54.1% and 45.9%, respectively). A high percentage of *very low*/*low* respondents (88.2%) commented that they did not have risk factors for stroke because they were young. **Table 2** shows the coding schema and distribution of responses.

Research Question 3: What are the most important factors for creating a message (type of message, source credibility, modality)? Does that differ by sex or age?

**Figure 1.**
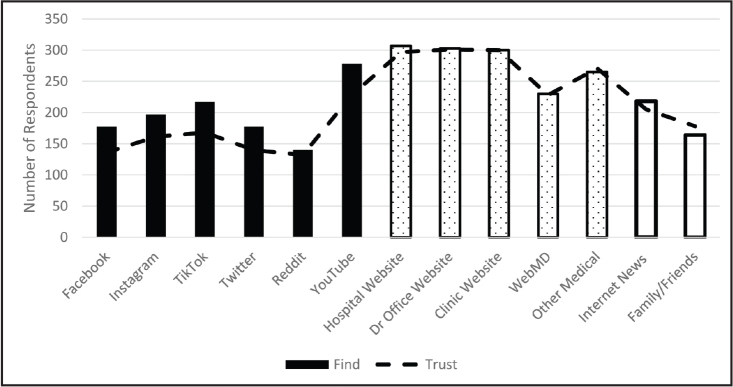
Finding and trusting health information. To cluster similar sources of health information together for further analysis, we created three categories. Social media sources are represented by solid black bars. Medical sources are represented by dotted bars. Other sources are represented by solid white bars.

**Figure 2.**
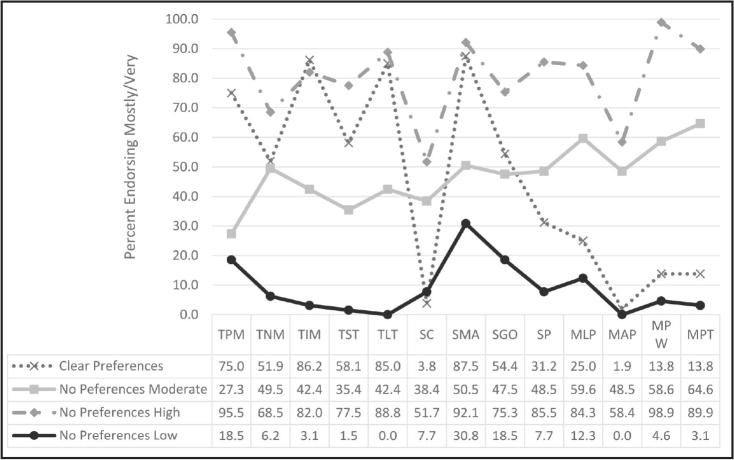
Message type, source credibility, and modality. MAP = modality – animated person; MLP = modality – living person; MPT = modality – pictures talking; MPW = modality – pictures written; SC = source – celebrity; SGO = source – government organization; SMA = source – medical authority; SP = source – people who look like me; TIM = type of message – informational; TLT = type of message – long-term; TNM = type of message – negative; TPM = type of message – positive; TST = type of message – short-term.

To address research question 3, we conducted a two-step cluster analysis with 13 variables to identify respondents' preferences of message type, credibility source, and delivery modality (**Figure 1**). A two-step cluster analysis was preferred because this approach empirically determined the optimal number of clusters based on different combinations of our 13 variables and we did not have an a priori number of clusters in mind. The results indicated that there were four distinct clusters (see **Figure 2**): Clear Preferences (38.7%, *n* = 160 ), No Preferences Moderate (24%, *n* = 99), No Preferences High (21.5%, *n* = 89), and No Preferences Low (15.7%, *n* = 65). The Clear Preferences cluster was most selective in choosing items. This indicates that this group had more preferences and differences in ratings compared to the other clusters that tended to respond more homogenously: No Preferences High chose *mostly*/*very* across all items, No Preferences Low chose *not at all*/*somewhat* across all items, No Preferences Moderate chose evenly between *high* and *low*. Clear Preferences results show higher scores on positive, informational, long-term messages and medical authority sources and lower scores on celebrity sources, animated person, pictures with writing, and pictures with talking modalities. There was no difference by sex or age.

Research Question 4: Where do people look for and trust health information? Does that differ by sex or age?

**Figure 3.**
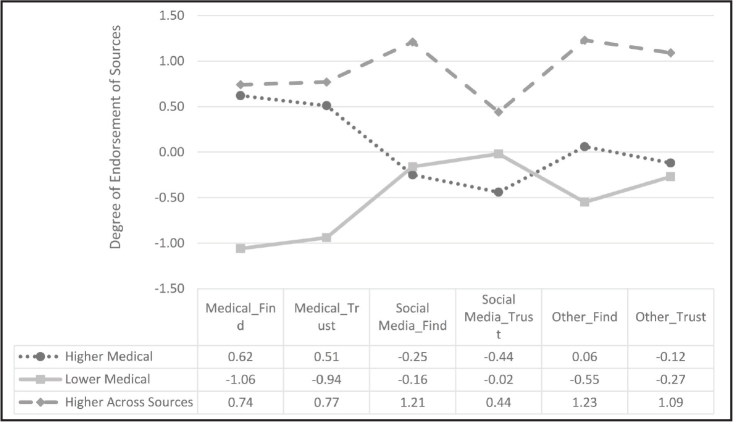
Finding and trusting health information sources. The y-axis represents z-scores (to put all health information sources on the same scale); 0 = mean and negative numbers indicate responses lower than the mean and positive numbers indicate responses higher than the mean.

To address research question 4, we conducted a two-step cluster analysis. Prior to running the analysis, we computed three *z*-scored scales for our finding health information source variables (medical, social media, and other) and three *z*-scored scales for our trusting health information source variables (medical, social media, and other; see **Figure 1** for all variables included in the scales). A two-step cluster analysis was preferred because this approach empirically determined the optimal number of clusters based on different combinations of our six scales and we did not have an a priori number of clusters in mind. The results indicated that there were three distinct clusters based on the six scales (see **Figure 3**). We descriptively labeled the clusters as: higher medical (47.2%, *n* = 195), lower medical (38%, *n* = 157), and higher across all sources (14.8%, *n* = 61) (**Figure 3**). There was no difference by sex or age.

## Discussion

Targeting young adult African Americans for stroke research is a way to increase awareness among this population, which is often understudied. Understanding how to create targeted health education messages for this cohort can promote stroke risk awareness and lead to effective and informed decision-making about health. Most respondents rated their perceived risk of stroke as *very low* or *low* and attributed being healthy, not having a family history of stroke, and youth to their rating. These results are not surprising considering the mean age (23.1 years) of respondents and similar findings observed in the literature (Aycock et al., 2017; Aycock et al., 2023). As this population ages and associated risk for stroke increase it is important to stress the significance of knowing one's personal risk of stroke and attaining and maintaining a healthy lifestyle. This sample was also less likely to identify specific disease and behavior-related factors as demonstrated by their moderate risk factor awareness scores.

The perceived risk of stroke was associated with risk factor awareness such that respondents who perceived their risk as moderate or higher had a greater awareness of stroke risk factors. Most respondents who perceived a higher risk of stroke also acknowledged having a risk factor for stroke (e.g., hypertension, obesity), which may explain this finding. In a review of the literature on the perceived risk of stroke among adults age 40 to 75 years, the most common predictor of a higher perceived risk of stroke was having a known or established risk factor for stroke (Aycock et al., 2017). Unhealthy diet, physical inactivity, and unhealthy social behaviors (i.e., smoking, alcohol consumption) common among young adults may not pose the same perceived risk ratings for stroke. Health messaging about lifestyle behaviors as potential risk factors for stroke is critical for young adults.

In our study, the largest cluster of respondents (*n* = 160) showed clear preferences in components of message creation (type of message, source credibility, modality); other respondent clusters provided homogenous responses across variables which may indicate no preference for messaging tactics. Designing effective tailored health communication campaigns can have significant influence on behavioral intentions to implement recommendations. Some literature shows that negative messages may lead to higher intentions for preventive health behaviors. The overriding tenet in message creation is to tailor the message to your audience (Kreuter & Wray, 2003); positive messaging was considered a more preferred messaging tactic by this cohort, which may indicate a focus on prevention behaviors (Keller & Lehmann, 2008). A meta-analysis of message communications and intentions show that focusing on changing unhealthy behavior through informational messages may also have significant impact on behavioral intention to change for targeted audiences (Keller and Lehmann, 2008). There was a clear preference for receiving health information from medical sources; most Americans trust health information from doctors more than any other source (Earnshaw et al., 2020; Jackson et al., 2019).

Understanding online health information seeking behavior (HISB) helps developers of health messages recognize where people find and trust health information. In our study, two-thirds of respondents had above average scores in both finding and trusting health information from medical sources. More than 85% of respondents had below average scores in finding and trusting health information from social media. The Comprehensive Model of Information Seeking posits three inherent variables to information seeking: antecedents (demographics, personal experience, salience, and beliefs), information carriers (sources, channels, message utility), actions (seeking or scanning information) (Johnson et al., 1995). Systematic reviews of HISB show conflicting results as to the greater importance of antecedents or information carriers (Wang et al., 2021); however, communication and dissemination strategies responding to higher levels of audience specificity may improve relevance in both message format and delivery for targeted audiences (Krueter & Wray, 2003; Ravenall et al., 2015; Schmid et al., 2008).

Findings from this study will help guide development of an educational intervention for young adult African Americans focusing on stroke risk awareness. We found that our study population was less aware of modifiable stroke risk factors such as unhealthy diet, physical inactivity, and unhealthy social behaviors (i.e., smoking, alcohol consumption). Our intent is to create an educational intervention that focuses on these modifiable behaviors rather than non-modifiable behaviors. Although preference for message modality (live person, animation, text, spoken word) was not significant, a positive message that was informational and showed a long-term result and was presented by a medical authority was clearly the highest-ranking preference. We did not observe any age or sex differences among the data, which suggests different message modalities by these demographics are not needed.

## Limitations

This study has several limitations. Data were collected through self-report, which can create social desirability bias in the responses and may affect validity of the responses. We only collected data from those who were able to access an online survey (convenience sampling), which means that those with low digital access and/or literacy may have been missed, leading to findings not being generalizable among the entire population of young adult African Americans in Georgia or variances being misrepresented. It is difficult to assess what percentage of our population cohort may have been excluded. Many people access the internet using broadband and others access the internet using cellular services; census data tells us that 9.9% of Georgians do not have internet access but these data are not disaggregated by age, thus we are unable to assess if low digital access significantly affects our cohort or our findings. The stroke risk awareness survey was not inclusive of all modifiable and non-modifiable stroke risks; therefore, comparisons to the perceived risk may have been underestimated. Despite this, stroke risk most common among this population were included and the measure had adequate internal consistency reliability.
